# Supplementary material for: geneRFinder: gene finding in distinct metagenomic data complexities
Source: BMC Bioinformatics. 2021 Feb 25;22:87. doi: 10.1186/s12859-021-03997-w (PMC7905635; doi:10.1186/s12859-021-03997-w)
Supplement: Supplementary file 1 — Additional file 1. Additional informationabout GeneRFinder-Benchmark and CAMI, genomes lists, contingency tables and repositories guides. [file 12859_2021_3997_MOESM1_ESM.pdf]

geneRFinder: gene finding in distinct metagenomic data complexities

Raíssa Silva, Kleber Padovani, Fabiana Góes and Ronnie Alves

1. Additional information about GeneRFinder-Benchmark

The ORFs extracted from CAMI genome assemblies have similar size distribution, with a predominance of sequences smaller than 200bp (Figure1). This is due to the fact that ORF extraction considered as ORF all sequences started with start codon and ended with some stop codon, regardless of its position. In this way, ORFs that were inside ORFs were also extracted. Thus, even though an ORF is part of the coding sequence, does not represent the entire gene, but only a fraction of it.

ORFs from the 12 annotated genomes of the first set of tests, named Test in Figure1, have a more balanced size distribution, however, ORFs under 200bp are also greater in this dataset. It is worth noting that the amount of ORFs less than 200bp is inversely proportional to the complexity of these samples. This is probably due to the fact that low complexity samples have, by definition, fewer genomes [1], that can reduce the complexity of the assembly process and thus obtaining larger and more reliable sequences, which will be used for the process of ORF extraction. This scenario may justify the increase in ORFs as complexity decreases.

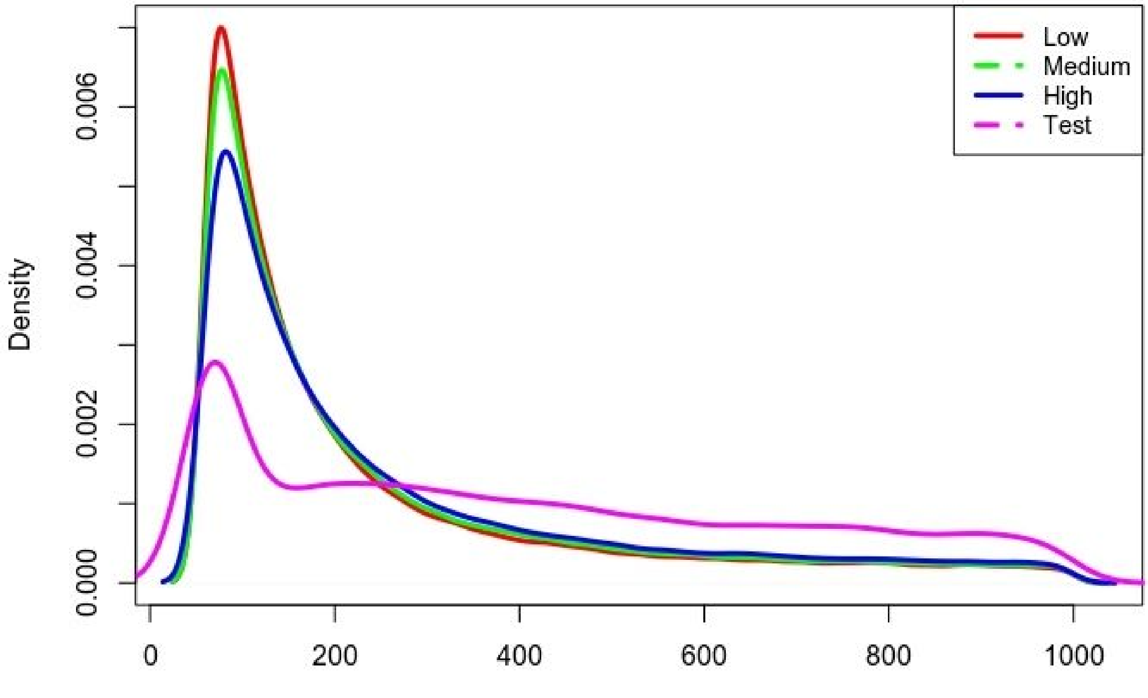

Figure 1. ORFs length distribution in the benchmark dataset.

2. Additional information about simulated genomes from CAMI

In order to increase strain level diversity, CAMI used 12 strain genomes that were simulated to produce the low complexity dataset. All information about related organisms and simulation process can be found at CAMI paper [2]. The names of the organisms of the simulated genomes mentioned are presented in the supplementary tables 5 to 9, using the same nomenclature of the genome files provided by CAMI.

Supplementary Table 1. List of genomes used in the initial training (training1)

Taxonomy_ID	Name	Rank
62977	Acinetobacter sp ADP1	species
272557	Aeropyrum pernix	species
264462	Bdellovibrio bacteriovorus	species
257310	Bordetella bronchiseptica	species
262698	Brucella abortus 9-941	species
195099	Campylobacter jejuni RM1221	species
203907	Candidatus Blochmannia floridanus	species
159087	Dechloromonas aromatica RCB	species
243164	Dehalococcoides ethenogenes 195	species
226185	Enterococcus faecalis V583	species
177416	Francisella tularensis tularensis	species
106370	Frankia casuarinae	species
251221	Gloeobacter violaceus	species
290633	Gluconobacter oxydans 621H	species
233412	Haemophilus ducreyi 35000HP	species
349521	Hahella chejuensis KCTC 2396	species
283942	Idiomarina loihiensis L2TR	species
272621	Lactobacillus acidophilus NCFM	species
272623	Lactococcus lactis	species
265311	Mesoplasma florum L1	species

Supplementary Table 2. List of genomes used in the validation (validation)

Taxonomy_ID	Name	Rank
348780	Natronomonas pharaonis	species
107806	Buchnera aphidicola	species

272560	Burkholderia pseudomallei	species
224308	Bacillus subtilis	species
306537	Corynebacterium jeikeium	species

Supplementary Table 3. List of genomes used in the full training (training2)

Taxonomy_ID	Name	Rank
272569	Haloarcula marismortui ATCC 43049 chromosome I	species
272569	Haloarcula marismortui ATCC 43049 chromosome II	species
64091	Halobacterium salinarum NRC-1	species
267377	Methanococcus maripaludis S2	species
188937	Methanosarcina acetivorans C2A	species
339860	Methanosphaera stadtmanae DSM 3091	species
187420	Methanothermobacter thermautotrophicus str. Delta H	species
263820	Picrophilus torridus DSM 9790	species
330779	Sulfolobus acidocaldarius DSM 639	species
69014	Thermococcus kodakarensis KOD1	species
273075	Thermoplasma acidophilum DSM 1728	species
62977	Acinetobacter sp. ADP1	species
176299	Agrobacterium tumefaciens str. C58 chromosome circular	species
176299	Agrobacterium tumefaciens str. C58 chromosome linear	species
234826	Anaplasma marginale str. St. Maries	species
224324	Aquifex aeolicus VF5	species
322098	Aster yellows witches'-broom phytoplasma AYWB	species
76114	Azoarcus sp. EbN1	species
272559	Bacteroides fragilis NCTC 9343	species
283166	Bartonella henselae str. Houston-1	species
264462	Bdellovibrio bacteriovorus HD100	species

206672	Bifidobacterium longum NCC2705	species
257310	Bordetella bronchiseptica RB50	species
224326	Borrelia burgdorferi B31	species
224911	Bradyrhizobium japonicum USDA 110	species
262698	Brucella abortus biovar 1 str. 9-941 chromosome I	species
262698	Brucella abortus biovar 1 str. 9-941 chromosome II	species
195099	Campylobacter jejuni RM1221	species
203907	Candidatus Blochmannia floridanus	species
246194	Carboxydothermus hydrogenoformans Z-2901	species
190650	Caulobacter crescentus CB15	species
243161	Chlamydia muridarum Nigg	species
218497	Chlamydophila abortus S26/3	species
243365	Chromobacterium violaceum ATCC 12472	species
272562	Clostridium acetobutylicum ATCC 824	species
167879	Colwellia psychrerythraea 34H	species
227377	Coxiella burnetii RSA 493	species
321327	Cyanobacteria bacterium Yellowstone A-Prime	species
159087	Dechloromonas aromatica RCB	species
243164	Dehalococcoides ethenogenes 195	species
243230	Deinococcus radiodurans R1	species
177439	Desulfotalea psychrophila LSv54	species
207559	Desulfovibrio desulfuricans G20	species
269484	Ehrlichia canis str. Jake	species
226185	Enterococcus faecalis V583	species
218491	Erwinia carotovora subsp. atroseptica SCRI1043	species
177416	Francisella tularensis subsp. tularensis SCHU S4	species
106370	Frankia casuarinae	species

190304	Fusobacterium nucleatum subsp. nucleatum ATCC 25586	species
235909	Geobacillus kaustophilus HTA426	species
269799	Geobacter metallireducens GS-15	species
251221	Gloeobacter violaceus PCC 7421	species
290633	Gluconobacter oxydans 621H	species
233412	Haemophilus ducreyi 35000HP	species
349521	Hahella chejuensis KCTC 2396	species
283942	Idiomarina loihiensis L2TR	species
272621	Lactobacillus acidophilus NCFM	species
272623	Lactococcus lactis subsp. lactis Il1403	species
297245	Legionella pneumophila str. Lens	species
281090	Leifsonia xyli subsp. xyli str. CTCB07	species
267671	Leptospira interrogans serovar Copenhageni str. Fiocruz L1-130 chromosome I	species
267671	Leptospira interrogans serovar Copenhageni str. Fiocruz L1-130 chromosome II	species
272626	Listeria innocua Clip11262	species
342108	Magnetospirillum magneticum AMB-1	species
221988	Mannheimia succiniciproducens MBEL55E	species
265311	Mesoplasma florum L1	species
266835	Mesorhizobium loti MAFF303099	species
243233	Methylococcus capsulatus str. Bath	species
264732	Moorella thermoacetica ATCC 39073	species
262316	Mycobacterium avium subsp. paratuberculosis K-10	species
340047	Mycoplasma capricolum subsp. capricolum ATCC 27343	species
242231	Neisseria gonorrhoeae FA 1090	species
323098	Nitrobacter winogradskyi Nb-255	species
323261	Nitrosococcus oceani ATCC 19707	species

228410	Nitrosomonas europaea ATCC 19718	species
323848	Nitrosospira multiformis ATCC 25196	species
247156	Nocardia farcinica IFM 10152	species
279238	Novosphingobium aromaticivorans DSM 12444	species
221109	Oceanobacillus iheyensis HTE831	species
262768	Onion yellows phytoplasma OY-M	species
272843	Pasteurella multocida subsp. multocida str. Pm70	species
338963	Pelobacter carbinolicus DSM 2380	species
319225	Pelodictyon luteolum DSM 273	species
298386	Photobacterium profundum SS9	species
298386	Photobacterium profundum SS9 chromosome 2	species
243265	Photorhabdus luminescens subsp. laumondii TTO1	species
242619	Porphyromonas gingivalis W83	species
267747	Propionibacterium acnes KPA171202	species
326442	Pseudoalteromonas haloplanktis TAC125 chromosome I	species
326442	Pseudoalteromonas haloplanktis TAC125 chromosome II	species
208964	Pseudomonas aeruginosa PAO1	species
259536	Psychrobacter arcticus 273-4	species
264198	Ralstonia eutropha JMP134 chromosome 1	species
264198	Ralstonia eutropha JMP134 chromosome 2	species
347834	Rhizobium etli CFN 42	species
272943	Rhodobacter sphaeroides 2.4.1 chromosome 1	species
272943	Rhodobacter sphaeroides 2.4.1 chromosome 2	species
243090	Rhodopirellula baltica SH 1	species
258594	Rhodopseudomonas palustris CGA009	species
269796	Rhodospirillum rubrum ATCC 11170	species
272944	Rickettsia conorii str. Malish 7	species

309807	Salinibacter ruber DSM 13855	species
321314	Salmonella enterica subsp. enterica serovar Choleraesuis str. SC-B67	species
211586	Shewanella oneidensis MR-1	species
300268	Shigella boydii Sb227	species
246200	Silicibacter (ncbi = Ruegeria) pomeroyi DSS-3	species
266834	Sinorhizobium meliloti 1021	species
343509	Sodalis glossinidius str. 'morsitans'	species
273036	Staphylococcus aureus RF122	species
208435	Streptococcus agalactiae 2603V/R	species
227882	Streptomyces avermitilis MA-4680	species
292459	Symbiobacterium thermophilum IAM 14863	species
1148	Synechocystis sp. PCC 6803	species
273068	Thermoanaerobacter tengcongensis MB4	species
269800	Thermobifida fusca YX	species
197221	Thermosynechococcus elongatus BP-1	species
243274	Thermotoga maritima MSB8	species
262724	Thermus thermophilus HB27	species
292415	Thiobacillus denitrificans ATCC 25259	species
317025	Thiomicrospira crunogena XCL-2	species
243275	Treponema denticola ATCC 35405	species
218496	Tropheryma whipplei TW08/27	species
273119	Ureaplasma parvum serovar 3 str. ATCC 700970	species
243277	Vibrio cholerae O1 biovar eltor str. N16961 chromosome I	species
243277	Vibrio cholerae O1 biovar eltor str. N16961 chromosome II	species
36870	Wigglesworthia glossinidia endosymbiont of Glossina brevipalpis	species
273121	Wolinella succinogenes DSM 1740	species

190486	Xanthomonas axonopodis pv. citri str. 306	species
160492	Xylella fastidiosa 9a5c	species

Supplementary Table 4. List of genomes used in the test (test1)

Taxonomy_ID	Name	Rank
243232	Methanocaldococcus jannaschii	species
224325	Archaeoglobus fulgidus	species
348780	Natronomonas pharaonis	species
83332	Mycobacterium tuberculosis	species
272560	Burkholderia pseudomallei	species
224308	Bacillus subtilis subsp. subtilis	species
306537	Corynebacterium jeikeium	species
194439	Chlorobium tepidum 0.8475 4	species
511145	Escherichia coli str. K-12	species
85963	Helicobacter pylori	species
74546	Prochlorococcus marinus	species
292805	Wolbachia endosymbiont	species

Supplementary Table 5. List of genomes used in the low complexity dataset (test2low)

Taxonomy_ID	Name	Rank
1123015	Pseudomonas aeruginosa	species
1121001	Andreprevotia lacus	species
1121885	Flavisolibacter ginsengiterrae	species
1122187	Lysobacter oryzae	species
644383	Thermosporothrix hazakensis	species
2088	Anaeroplasma bactoclasticum	species
1121884	Flavisolibacter ginsengisoli	species
1123243	Schwartzia succinivorans	species

1122156	Lampropedia hyalina	species
1123349	Tepidibacter formicigenes	species
1121301	Paramaledivibacter caminithermalis	species
1120989	Anaerobranca californiensis	species
1120996	Anaerosporobacter mobilis	species
1121393	Desulfatibacillum alkenivorans	species
2371	Xylella fastidiosa	species
328515	Nonlabens dokdonensis	species
404881	Defluviimonas denitrificans	species
1004304	Hydrotalea sandarakina	species
990712	Albidovulum xiamenense	species
266	Paracoccus denitrificans	species
266	Paracoccus denitrificans	species
266	Paracoccus denitrificans	species
29580	Janthinobacterium	genus
173053	Tetrasphaera duodecadis	species
75309	Rhodanobacter	genus
2037	Actinomycetales	order
1385	Bacillales	order
29580	Janthinobacterium	genus
2088	evo_1035930.011	strain
2088	evo_1035930.029	strain
2088	evo_1035930.032	strain
1004304	evo_1049056.011	strain
1004304	evo_1049056.013	strain
1004304	evo_1049056.015	strain
1004304	evo_1049056.031	strain

1004304	evo_1049056.039	strain
1385	evo_1286_AP.008	strain
1385	evo_1286_AP.026	strain
1385	evo_1286_AP.033	strain
1385	evo_1286_AP.037	strain

Supplementary Table 6. List of genomes used in the medium complexity dataset (test2medium)

Taxonomy_ID	Name	Rank
1763	Mycobacterium	genus
80864	Comamonadaceae	family
80864	Comamonadaceae	family
2062	Streptomycetaceae	family
1707	Cellulomonas	genus
1121911	Garciella nitratireducens	species
1121270	Carboxydocella sporoproducens	species
1123491	Vibrio cincinnatiensis	species
1121442	Desulfovibrio bizertensis	species
1123232	Salinicoccus kunmingensis	species
1123358	Tetragenococcus halophilus	species
1120981	Alysiella filiformis	species
1121478	Diaphorobacter oryzae	species
1121466	Desulfovibrio vietnamensis	species
1122961	Picrophilus oshimae	species
1122958	Phenylobacterium composti	species
1123266	Sphingomonas aestuarii	species
1121439	Desulfovibrio alkalitolerans	species
1123492	Vibrio gazogenes	species

39498	[Eubacterium] yurii	species
745369	Acetoanaerobium noterae	species
35623	Acholeplasma oculi	species
915	Nitrosomonas europaea	species
466	Legionella maceachernii	species
393921	Porphyromonas crevioricanis	species
1513	Clostridium tetani	species
28136	Prevotella oulorum	species
1122184	Lutispora thermophila	species
1121088	Bacillus coagulans	species
1121881	Ferrithrix thermotolerans	species
1121264	Caloranaerobacter azorensis	species
1121128	Butyricicoccus pullicaecorum	species
1121316	Clostridium grantii	species
1121131	Butyrivibrio fibrisolvens	species
1121132	Butyrivibrio hungatei	species
1123350	Tepidibacter thalassicus	species
1123003	Propionispora hippei	species
1121428	Desulfotomaculum hydrothermale	species
1121266	Caminicella sporogenes	species
445973	Intestinibacter bartlettii	species
299255	Ferrimonas marina	species
1123357	Tessaracoccus bendigoensis	species
1123029	Pseudoxanthobacter soli	species
103732	Lechevalieria flava	species
68170	Lechevalieria aerocolonigenes	species
2030	Kibdelosporangium aridum	species

639310	Olleya aquimaris	species
688913	Ohtaekwangia kribbensis	species
688867	Ohtaekwangia koreensis	species
1061	Rhodobacter capsulatus	species
121821	Roseinatronobacter thiooxidans	species
441209	Rhodobaca barguzinensis	species
561061	Sphingobacterium psychroaquaticum	species
570520	Formosa spongicola	species
1513896	Sphingobacterium nematocida	species
354	Azotobacter vinelandii	species
82367	Paracoccus pantotrophus	species
82367	Paracoccus pantotrophus	species
2242	Halobacterium salinarum	species
2242	Halobacterium salinarum	species
1061	Rhodobacter capsulatus	species
759851	Sporosarcina newyorkensis	species
759851	Sporosarcina newyorkensis	species
382	Sinorhizobium meliloti	species
382	Sinorhizobium meliloti	species
1525	Moorella thermoacetica	species
1525	Moorella thermoacetica	species
1082	Phaeospirillum fulvum	species
1082	Phaeospirillum fulvum	species
1082	Phaeospirillum fulvum	species
1082	Phaeospirillum fulvum	species
1516	Thermoanaerobacter thermohydrosulfuricus	species
1516	Thermoanaerobacter thermohydrosulfuricus	species

192	Azospirillum brasilense	species
629679	Promicromonospora umidemergens	species
280	Xanthobacter autotrophicus	species
69278	Aquamicrobium	genus
85413	Bosea	genus
379	Rhizobium	genus
13687	Sphingomonas	genus
80864	Comamonadaceae	family
162491	Oerskovia	genus
80864	Comamonadaceae	family
1707	Cellulomonas	genus
80864	Comamonadaceae	family
13687	Sphingomonas	genus
169973	uncultured Massilia sp.	species
1143711	Paenibacillus frigoriresistens	species
367298	Phycicoccus	genus
1707	Cellulomonas	genus
85023	Microbacteriaceae	family
13687	Sphingomonas	genus
169973	uncultured Massilia sp.	species
69278	Aquamicrobium	genus
1817	Nocardia	genus
53458	Janibacter limosus	species
460257	Aeromicrobium ponti	species
1827	Rhodococcus	genus
311234	Cellulomonas terrae	species
59732	Chryseobacterium	genus

1707	Cellulomonas	genus
460257	Aeromicrobium ponti	species
16	Methylophilus	genus
92511	Curtobacterium sp. B20	species
257003	Sphingomonas phyllosphaerae	species
460257	Aeromicrobium ponti	species
454586	Pedobacter agri	species
255475	Aurantimonadaceae	family
16	Methylophilus	genus
16	Methylophilus	genus
85413	Bosea	genus
1385	Bacillales	order
255475	Aurantimonadaceae	family
2062	Streptomycetaceae	family
367298	Phycicoccus	genus
84567	Pedobacter	genus
34038	Rahnella aquatilis	species
162491	Oerskovia	genus
80864	Comamonadaceae	family
1121270	evo_1030728.001	strain
1121270	evo_1030728.009	strain
1121270	evo_1030728.011	strain
1121270	evo_1030728.035	strain
1121270	evo_1030728.038	strain
745369	evo_1035921.007	strain
745369	evo_1035921.008	strain
745369	evo_1035921.028	strain

745369	evo_1035921.030	strain
169973	evo_1139_Y.018	strain
169973	evo_1139_Y.035	strain
1385	evo_1286_G.010	strain
1385	evo_1286_G.034	strain

Supplementary Table 7. List of genomes used in the high complexity dataset, sample 1 (test2high1)

Taxonomy_ID	Name	Rank
478009	Halobacterium salinarum	species
2037	Actinomycetales	order
1763	Mycobacterium	genus
1817	Nocardia	genus
756689	Nocardia amikacinitolerans	species
1827	Rhodococcus	genus
908626	Angustibacter	genus
1707	Cellulomonas	genus
162491	Oerskovia	genus
53458	Janibacter limosus	species
136099	Knoellia	genus
367298	Phycicoccus	genus
53355	Terrabacter	genus
85023	Microbacteriaceae	family
33877	Agromyces	genus
293890	Agromyces subbeticus	species
2034	Curtobacterium	genus
96492	Frigoribacterium	genus
110932	Leifsonia	genus

33882	Microbacterium	genus
190323	Plantibacter	genus
33886	Rathayibacter	genus
1663	Arthrobacter	genus
60919	Sanguibacter	genus
2040	Aeromicrobium	genus
1839	Nocardioides	genus
1843	Nocardioides jensenii	species
42197	Actinosynnema pretiosum	species
2030	Kibdelosporangium aridum	species
68170	Lechevalieria aerocolonigenes	species
40571	Lentzea albidocapillata	species
1933	Streptoalloteichus tenebrarius	species
2062	Streptomycetaceae	family
2063	Kitasatospora	genus
1883	Streptomyces	genus
36874	Porphyromonas cangingivalis	species
393921	Porphyromonas crevioricanis	species
1122991	Prevotella shahii	species
1004	Chitinophaga sancti	species
279824	Algoriphagus alkaliphilus	species
990	Cytophaga xylanolytica	species
292407	Dyadobacter crusticola	species
76595	Cellulophaga fucicola	species
59732	Chryseobacterium	genus
172045	Elizabethkingia miricola	species
237	Flavobacterium	genus

49280	Gelidibacter algens	species
328515	Nonlabens dokdonensis	species
906888	Nonlabens ulvanivorans	species
270918	Salegentibacter mishustinae	species
435906	Salegentibacter salarius	species
84567	Pedobacter	genus
454586	Pedobacter agri	species
188932	Pedobacter cryoconitis	species
1385	Bacillales	order
1386	Bacillus	genus
1121088	Bacillus coagulans	species
55080	Brevibacillus	genus
44249	Paenibacillus	genus
684063	Paenibacillus algorifonticola	species
1123358	Tetragenococcus halophilus	species
1122148	Lactobacillus lindneri	species
1122150	Lactobacillus nagelii	species
1122151	Lactobacillus paralimentarius	species
1121128	Butyricicoccus pullicaecorum	species
1121320	Clostridium intestinale	species
1121326	Clostridium magnum	species
1513	Clostridium tetani	species
42322	Eubacterium ruminantium	species
1121131	Butyrivibrio fibrisolvens	species
1121132	Butyrivibrio hungatei	species
1123012	Pseudobutyrivibrio xylanivorans	species
1121421	Desulfotomaculum aeronauticum	species

1121428	Desulfotomaculum hydrothermale	species
445973	Intestinibacter bartlettii	species
1121324	Peptoclostridium litorale	species
1525	Moorella thermoacetica	species
1516	Thermoanaerobacter thermohydrosulfuricus	species
1120997	Anaerovibrio lipolyticus	species
349095	Megasphaera paucivorans	species
41275	Brevundimonas	genus
75	Caulobacter	genus
255475	Aurantimonadaceae	family
414371	Aureimonas	genus
1121026	Aureimonas altamirensis	species
85413	Bosea	genus
53254	Bosea thiooxidans	species
374	Bradyrhizobium	genus
46913	Devosia	genus
1121477	Devosia limi	species
407	Methylobacterium	genus
1122234	Methylobacterium komagatae	species
31988	Aminobacter	genus
561088	Aquamicrobium aerolatum	species
68287	Mesorhizobium	genus
245876	Nitratireductor	genus
106591	Ensifer	genus
379	Rhizobium	genus
382	Sinorhizobium meliloti	species
1080	Afifella marina	species

280	Xanthobacter autotrophicus	species
990712	Albidovulum xiamenense	species
576117	Celeribacter halophilus	species
402884	Cereibacter changlensis	species
561184	Mameliella alba	species
266	Paracoccus denitrificans	species
82367	Paracoccus pantotrophus	species
1061	Rhodobacter capsulatus	species
121821	Roseinatronobacter thiooxidans	species
266809	Thalassobacter stenotrophicus	species
1120923	Acidocella aminolytica	species
192	Azospirillum brasilense	species
1082	Phaeospirillum fulvum	species
361177	Altererythrobacter	genus
198312	Porphyrobacter sanguineus	species
165695	Sphingobium	genus
13687	Sphingomonas	genus
563996	Sphingomonas hankookensis	species
1123269	Sphingomonas sanxanigenens	species
160791	Sphingomonas wittichii	species
165697	Sphingopyxis	genus
506	Alcaligenaceae	family
80864	Comamonadaceae	family
12916	Acidovorax	genus
747294	Pseudorhodoferax	genus
174951	Ramlibacter	genus
34072	Variovorax	genus

75654	Duganella	genus
29580	Janthinobacterium	genus
149698	Massilia	genus
16	Methylophilus	genus
915	Nitrosomonas europaea	species
1121029	Azoarcus communis	species
1121393	Desulfatibacillum alkenivorans	species
1121405	Desulfococcus multivorans	species
91360	Desulforhopalus singaporensis	species
1121439	Desulfovibrio alkalitolerans	species
876	Desulfovibrio desulfuricans	species
1123324	Succinivibrio dextrinosolvens	species
299255	Ferrimonas marina	species
543	Enterobacteriaceae	family
1123238	Atlantibacter subterranea	species
1121872	Erwinia tracheiphila	species
53335	Pantoea	genus
469	Acinetobacter	genus
170623	Azotobacter beijerinckii	species
354	Azotobacter vinelandii	species
286	Pseudomonas	genus
1123015	Pseudomonas aeruginosa	species
1123022	Pseudomonas xiamenensis	species
75309	Rhodanobacter	genus
32033	Xanthomonadaceae	family
1121014	Arenimonas donghaensis	species
68	Lysobacter	genus

1122185	Lysobacter concretionis	species
83618	Pseudoxanthomonas	genus
40323	Stenotrophomonas	genus
338	Xanthomonas	genus
2371	Xylella fastidiosa	species
48467	Prosthecobacter debontii	species

Supplementary Table 8. List of genomes used in the high complexity dataset, sample 2 (test2high2)

Taxonomy_ID	Name	Rank
478009	Halobacterium salinarum	species
2037	Actinomycetales	order
1763	Mycobacterium	genus
1817	Nocardia	genus
756689	Nocardia amikacinitolerans	species
1827	Rhodococcus	genus
908626	Angustibacter	genus
1707	Cellulomonas	genus
162491	Oerskovia	genus
136099	Knoellia	genus
367298	Phycicoccus	genus
53355	Terrabacter	genus
85023	Microbacteriaceae	family
33877	Agromyces	genus
293890	Agromyces subbeticus	species
2034	Curtobacterium	genus
96492	Frigoribacterium	genus
110932	Leifsonia	genus

33882	Microbacterium	genus
190323	Plantibacter	genus
33886	Rathayibacter	genus
1663	Arthrobacter	genus
60919	Sanguibacter	genus
2040	Aeromicrobium	genus
1839	Nocardioides	genus
1843	Nocardioides jensenii	species
42197	Actinosynnema pretiosum	species
2030	Kibdelosporangium aridum	species
68170	Lechevalieria aerocolonigenes	species
40571	Lentzea albidocapillata	species
1933	Streptoalloteichus tenebrarius	species
2062	Streptomycetaceae	family
2063	Kitasatospora	genus
1883	Streptomyces	genus
36874	Porphyromonas cangingivalis	species
393921	Porphyromonas crevioricanis	species
1122991	Prevotella shahii	species
1004	Chitinophaga sancti	species
279824	Algoriphagus alkaliphilus	species
292407	Dyadobacter crusticola	species
76595	Cellulophaga fucicola	species
59732	Chryseobacterium	genus
172045	Elizabethkingia miricola	species
237	Flavobacterium	genus
49280	Gelidibacter algens	species

328515	Nonlabens dokdonensis	species
906888	Nonlabens ulvanivorans	species
270918	Salegentibacter mishustinae	species
435906	Salegentibacter salarius	species
84567	Pedobacter	genus
454586	Pedobacter agri	species
188932	Pedobacter cryoconitis	species
1385	Bacillales	order
1386	Bacillus	genus
1121088	Bacillus coagulans	species
55080	Brevibacillus	genus
44249	Paenibacillus	genus
684063	Paenibacillus algorifonticola	species
759851	Sporosarcina newyorkensis	species
1123358	Tetragenococcus halophilus	species
1122148	Lactobacillus lindneri	species
1122150	Lactobacillus nagelii	species
1122151	Lactobacillus paralimentarius	species
1121128	Butyricicoccus pullicaecorum	species
1121264	Caloranaerobacter azorensis	species
1121320	Clostridium intestinale	species
1121326	Clostridium magnum	species
1513	Clostridium tetani	species
42322	Eubacterium ruminantium	species
1121131	Butyrivibrio fibrisolvens	species
1121132	Butyrivibrio hungatei	species
1123012	Pseudobutyrivibrio xylanivorans	species

1121428	Desulfotomaculum hydrothermale	species
1121324	Peptoclostridium litorale	species
1525	Moorella thermoacetica	species
1120997	Anaerovibrio lipolyticus	species
349095	Megasphaera paucivorans	species
41275	Brevundimonas	genus
75	Caulobacter	genus
255475	Aurantimonadaceae	family
414371	Aureimonas	genus
1121026	Aureimonas altamirensis	species
85413	Bosea	genus
53254	Bosea thiooxidans	species
374	Bradyrhizobium	genus
46913	Devosia	genus
1121477	Devosia limi	species
407	Methylobacterium	genus
31988	Aminobacter	genus
561088	Aquamicrobium aerolatum	species
68287	Mesorhizobium	genus
245876	Nitratireductor	genus
106591	Ensifer	genus
379	Rhizobium	genus
382	Sinorhizobium meliloti	species
1080	Afifella marina	species
990712	Albidovulum xiamenense	species
576117	Celeribacter halophilus	species
561184	Mameliella alba	species

82367	Paracoccus pantotrophus	species
441209	Rhodobaca barguzinensis	species
1061	Rhodobacter capsulatus	species
121821	Roseinatronobacter thiooxidans	species
266809	Thalassobacter stenotrophicus	species
1120923	Acidocella aminolytica	species
192	Azospirillum brasilense	species
1082	Phaeospirillum fulvum	species
361177	Altererythrobacter	genus
198312	Porphyrobacter sanguineus	species
165695	Sphingobium	genus
13687	Sphingomonas	genus
563996	Sphingomonas hankookensis	species
257003	Sphingomonas phyllosphaerae	species
1123269	Sphingomonas sanxanigenens	species
160791	Sphingomonas wittichii	species
165697	Sphingopyxis	genus
506	Alcaligenaceae	family
80864	Comamonadaceae	family
12916	Acidovorax	genus
747294	Pseudorhodoferax	genus
174951	Ramlibacter	genus
34072	Variovorax	genus
75654	Duganella	genus
29580	Janthinobacterium	genus
149698	Massilia	genus
16	Methylophilus	genus

915	Nitrosomonas europaea	species
1121029	Azoarcus communis	species
1121405	Desulfococcus multivorans	species
91360	Desulforhopalus singaporensis	species
1121439	Desulfovibrio alkalitolerans	species
876	Desulfovibrio desulfuricans	species
1123324	Succinivibrio dextrinosolvens	species
543	Enterobacteriaceae	family
1123238	Atlantibacter subterranea	species
1121872	Erwinia tracheiphila	species
53335	Pantoea	genus
34038	Rahnella aquatilis	species
469	Acinetobacter	genus
170623	Azotobacter beijerinckii	species
354	Azotobacter vinelandii	species
286	Pseudomonas	genus
1123015	Pseudomonas aeruginosa	species
75309	Rhodanobacter	genus
32033	Xanthomonadaceae	family
1121014	Arenimonas donghaensis	species
68	Lysobacter	genus
1122185	Lysobacter concretionis	species
83618	Pseudoxanthomonas	genus
40323	Stenotrophomonas	genus
338	Xanthomonas	genus
2371	Xylella fastidiosa	species
35623	Acholeplasma oculi	species

171291	Mycoplasma verecundum	species
48467	Prosthecobacter debontii	species
48464	Prosthecobacter fusiformis	species

Supplementary Table 9. List of genomes used in the high complexity dataset, sample 3 (test2high3 dataset)

Taxonomy_ID	Name	Rank
478009	Halobacterium salinarum	species
2037	Actinomycetales	order
1763	Mycobacterium	genus
1817	Nocardia	genus
756689	Nocardia amikacinitolerans	species
1827	Rhodococcus	genus
908626	Angustibacter	genus
1707	Cellulomonas	genus
162491	Oerskovia	genus
136099	Knoellia	genus
367298	Phycicoccus	genus
53355	Terrabacter	genus
85023	Microbacteriaceae	family
33877	Agromyces	genus
293890	Agromyces subbeticus	species
2034	Curtobacterium	genus
96492	Frigoribacterium	genus
110932	Leifsonia	genus
33882	Microbacterium	genus
190323	Plantibacter	genus
33886	Rathayibacter	genus

1663	Arthrobacter	genus
60919	Sanguibacter	genus
2040	Aeromicrobium	genus
1839	Nocardioides	genus
1843	Nocardioides jensenii	species
42197	Actinosynnema pretiosum	species
2030	Kibdelosporangium aridum	species
68170	Lechevalieria aerocolonigenes	species
40571	Lentzea albidocapillata	species
1933	Streptoalloteichus tenebrarius	species
2062	Streptomycetaceae	family
2063	Kitasatospora	genus
1883	Streptomyces	genus
36874	Porphyromonas cangingivalis	species
28136	Prevotella oulorum	species
1122991	Prevotella shahii	species
1004	Chitinophaga sancti	species
279824	Algoriphagus alkaliphilus	species
292407	Dyadobacter crusticola	species
76595	Cellulophaga fucicola	species
59732	Chryseobacterium	genus
172045	Elizabethkingia miricola	species
237	Flavobacterium	genus
49280	Gelidibacter algens	species
328515	Nonlabens dokdonensis	species
906888	Nonlabens ulvanivorans	species
270918	Salegentibacter mishustinae	species

435906	Salegentibacter salarius	species
84567	Pedobacter	genus
454586	Pedobacter agri	species
188932	Pedobacter cryoconitis	species
1385	Bacillales	order
1386	Bacillus	genus
1121088	Bacillus coagulans	species
55080	Brevibacillus	genus
44249	Paenibacillus	genus
684063	Paenibacillus algorifonticola	species
759851	Sporosarcina newyorkensis	species
1122148	Lactobacillus lindneri	species
1122150	Lactobacillus nagelii	species
1122151	Lactobacillus paralimentarius	species
1121128	Butyricicoccus pullicaecorum	species
1121264	Caloranaerobacter azorensis	species
1121320	Clostridium intestinale	species
1121326	Clostridium magnum	species
1513	Clostridium tetani	species
42322	Eubacterium ruminantium	species
1121131	Butyrivibrio fibrisolvens	species
1121132	Butyrivibrio hungatei	species
1123012	Pseudobutyrivibrio xylanivorans	species
1121428	Desulfotomaculum hydrothermale	species
1121324	Peptoclostridium litorale	species
1525	Moorella thermoacetica	species
1516	Thermoanaerobacter thermohydrosulfuricus	species

1120997	Anaerovibrio lipolyticus	species
349095	Megasphaera paucivorans	species
41275	Brevundimonas	genus
75	Caulobacter	genus
255475	Aurantimonadaceae	family
414371	Aureimonas	genus
1121026	Aureimonas altamirensis	species
85413	Bosea	genus
53254	Bosea thiooxidans	species
374	Bradyrhizobium	genus
46913	Devosia	genus
1121477	Devosia limi	species
407	Methylobacterium	genus
31988	Aminobacter	genus
561088	Aquamicrobium aerolatum	species
68287	Mesorhizobium	genus
245876	Nitratireductor	genus
106591	Ensifer	genus
379	Rhizobium	genus
382	Sinorhizobium meliloti	species
1080	Afifella marina	species
280	Xanthobacter autotrophicus	species
990712	Albidovulum xiamenense	species
576117	Celeribacter halophilus	species
561184	Mameliella alba	species
441209	Rhodobaca barguzinensis	species
1061	Rhodobacter capsulatus	species

121821	Roseinatronobacter thiooxidans	species
266809	Thalassobacter stenotrophicus	species
1120923	Acidocella aminolytica	species
192	Azospirillum brasilense	species
1082	Phaeospirillum fulvum	species
361177	Altererythrobacter	genus
165695	Sphingobium	genus
13687	Sphingomonas	genus
563996	Sphingomonas hankookensis	species
257003	Sphingomonas phyllosphaerae	species
1123269	Sphingomonas sanxanigenens	species
160791	Sphingomonas wittichii	species
165697	Sphingopyxis	genus
1123272	Sphingorhabdus marina	species
506	Alcaligenaceae	family
80864	Comamonadaceae	family
12916	Acidovorax	genus
747294	Pseudorhodoferax	genus
174951	Ramlibacter	genus
34072	Variovorax	genus
75654	Duganella	genus
29580	Janthinobacterium	genus
149698	Massilia	genus
16	Methylophilus	genus
915	Nitrosomonas europaea	species
1121029	Azoarcus communis	species
1121393	Desulfatibacillum alkenivorans	species

1121405	Desulfococcus multivorans	species
91360	Desulforhopalus singaporensis	species
1121439	Desulfovibrio alkalitolerans	species
876	Desulfovibrio desulfuricans	species
1121391	Desulfacinum infernum	species
1123324	Succinivibrio dextrinosolvens	species
299255	Ferrimonas marina	species
543	Enterobacteriaceae	family
1121872	Erwinia tracheiphila	species
53335	Pantoea	genus
1121119	Brenneria alni	species
34038	Rahnella aquatilis	species
469	Acinetobacter	genus
170623	Azotobacter beijerinckii	species
354	Azotobacter vinelandii	species
286	Pseudomonas	genus
1123015	Pseudomonas aeruginosa	species
92487	Thiothrix eikelboomii	species
75309	Rhodanobacter	genus
32033	Xanthomonadaceae	family
1121014	Arenimonas donghaensis	species
68	Lysobacter	genus
1122185	Lysobacter concretionis	species
83618	Pseudoxanthomonas	genus
40323	Stenotrophomonas	genus
338	Xanthomonas	genus
2371	Xylella fastidiosa	species

48467	Prosthecobacter debontii	species

Supplementary Table 10. Contingency table between FragGeneScan (FG), Prodigal (PD) and geneRFinder (GF) in test dataset (test1)

	FG x GF		FG x PD		FG x PD	
	Positive	Negative	Positive	Negative	Positive	Negative
Positive	36270	14129	37287	1971	37264	1994
Negative	2316	2265	1299	14423	13135	2587
p-value: 0.00000000						

Supplementary Table 11. Contingency table between FragGeneScan (FG), Prodigal (PD) and geneRFinder (GF) in low dataset (test2low)

	FG x GF		FG x PD		FG x PD	
	Positive	Negative	Positive	Negative	Positive	Negative
Positive	75206	163524	70644	31888	99671	2861
Negative	2190	14669	6752	146305	139059	13998
p-value: 0.00000000						

Supplementary Table 12. Contingency table between FragGeneScan (FG), Prodigal (PD) and geneRFinder (GF) in medium dataset (test2medium)

	FG x GF		FG x PD		FG x PD	
	Positive	Negative	Positive	Negative	Positive	Negative
Positive	99974	226161	93883	45675	135274	4284
Negative	3182	18325	9273	198811	190861	17223
p-value: 0.00000000						

Supplementary Table 13. Contingency table between FragGeneScan (FG), Prodigal (PD) and geneRFinder (GF) in high dataset, sample 01 (test2high1)

	FG x GF		FG x PD		FG x PD	
	Positive	Negative	Positive	Negative	Positive	Negative

Positive	56651	130405	53743	26327	77482	2588
Negative	1851	11093	4759	115171	109574	10356
p-value: 0.00000000						

Supplementary Table 14. Contingency table between FragGeneScan (FG), Prodigal (PD) and geneRFinder (GF) in high dataset, sample 02 (test2high2)

	FG x GF		FG x PD		FG x PD	
	Positive	Negative	Positive	Negative	Positive	Negative
Positive	56311	130694	53433	25445	76377	2501
Negative	1829	11166	4707	116415	110628	10494
p-value: 0.00000000						

Supplementary Table 15. Contingency table between FragGeneScan (FG), Prodigal (PD) and geneRFinder (GF) in high dataset, sample 03 (test2high3)

	FG x GF		FG x PD		FG x PD	
	Positive	Negative	Positive	Negative	Positive	Negative
Positive	56336	130460	53442	25688	76460	2670
Negative	1905	11299	4799	116071	110336	10534
p-value: 0.00000000						

OSF Guide

Repository to reproduce the results of this paper

Link: https://osf.io/g4qk5/

1. Benchmark of Prediction Genes

In this folder are all the scripts and results generated to produce the benchmark from CAMI datasets.

1.1. fastas

FASTA with sequences belonging to ORFs extracted from CAMI datasets and returned from CD-HIT.

1.2. interpro

This folder contains:

- original_output: the original files outputted by InterproScan
- ipr: script to extract ORF ids which have associated IPR and the corresponding IDs extracted

1.3. model

This folder contains the RF model trained (saved as a caret object - in R).

1.4. script

This folder contains 5 scripts to reproduce paper experiments with CAMI separated by complexity as follows:

- Rscript test_low.R
- Rscript test_medium.R
- Rscript test_highS01.R
- Rscript test_highS02.R
- Rscript test_highS03.R

Additionally, it contains one script to extract ORFs from genomes. To execute it, it's required to update values for the two following variables inside the code:

- inputFile: Path of FASTA file with the corresponding genome
- outputFile: Path of the output FASTA with sequences of ORFs found in the input file

1.5. tableFeatures

This folder contains one file for each complexity. Each file has all ORFs extracted from original sample along with their features and corresponding labels. ORFs whose IDs were contained in InterproScan output were labeled as positive instance.

1.6. tableSequences

This folder contains auxiliary files that store the genomic content of each ORF extracted.

2. GeneRFinder

In this folder are all the implemented scripts and test results of the geneRFinder tool, as well as the results of the Prodigal and FragGeneScan tools.

2.1. modelTrain.R

Script to run train and make a new model.

2.2. PaperPrediction.Rproj

Gene prediction project.

2.3. predictionGenes.R

Script to predict genes.

2.4. src

In this folder can be found the scripts used to predict genes, make models and do tests:

- dataPreProcessing.R: Script to preprocess data
- extractSequences.R: Script to extract sequences
- functions.R: Script which contains all functions used
- getTableFeatures.R: Script to get a table with features from data
- modelGenerating.R: Script to run train and test scripts
- PaperPrediction.Rproj: Gene prediction project
- prediction.R: Script to predict genes
- readFastaFile.R: Script to read FASTA file.
- statistics: folder containing all tests to calculate the results, including the MCNemar test.

2.5. input

Files to produce test (test1) and training (training2) dataset. This folder contains:

- test: Folder with annotations for 12 organisms (complete genome, FASTA file and table features)
- train: Folder with link to download the annotations for 129 organisms (complete genome, FASTA file and table features)

2.6. output

Folder with results. In this folder can be found:

- cami: Table features and table sequences from FASTAS (section 1.1)
- FragGeneScan: Files from FragGeneScan results
- model: Output of training1 dataset
- predict: Predictions of the tools
- Prodigal: Files from Prodigal results
- test: Output of test1 dataset

3. Supplementary Data

This folder containing this document.

geneRFinder-Benchmark

Repository to find the geneRFinder benchmark

Link: https://sourceforge.net/projects/generfinder-benchmark/

The GeneRFinder-Benchmark is a comprehensive benchmark data for gene prediction which is based on data of CAMI (Critical Assessment of Metagenome Interpretation) and contains labeled data from gene regions.

The benchmark is made up of 9 datasets. For each one of them is provided:

- List of names, taxonomy ID and taxonomic level of the genomes of the organisms that make up the dataset (genomes.csv)
- Set of ORFs extracted from the respective selected genomes (sequences.fasta)
- Ground truth for each of the extracted ORFs (groundtruth.csv)

For information about download, see the documentation.

geneRFinder
Repository to find the geneRFinder tool
Link: https://github.com/railorena/geneRFinder

Installation
To install geneRFinder, please running the script:

```
Rscript ./src/config.R
```

Usage

```
Rscript ./geneRFinder.R -i [fasta_file_name] -o [output_file_name] -t [thread_number]
-s [start_type] -n [intergenic]
```

[fasta_file_name]: input file name
[output_file_name]: output file name
[thread_number]: number of thread
[start_type]:
1 - if start codon is ATG
2 - if start codon is ATG, GTG and TTG

[intergenic]:
1 - output without intergenic sequences
2 - output with intergenic sequences

For example,

```
Rscript ./geneRFinder.R -i ./example/final.contigs.fa -o output -t 7 -s 1 -n 1
```

Please, download the src/model.RData file separately, it is a large file.

References

1. National Research Council. (2007). The new science of metagenomics: revealing the secrets of our microbial planet. National Academies Press

2. Sczyrba, A., Hofmann, P., Belmann, P., Koslicki, D., Janssen, S., Dröge, J., ... & Bremges, A. (2017). Critical assessment of metagenome interpretation—a benchmark of metagenomics software. Nature methods, 14(11), 1063-1071.
